# Recent Developments in Compact Membrane Reactors with Hydrogen Separation

**DOI:** 10.3390/membranes8040107

**Published:** 2018-11-14

**Authors:** Alexander Wunsch, Paul Kant, Marijan Mohr, Katja Haas-Santo, Peter Pfeifer, Roland Dittmeyer

**Affiliations:** Institute for Micro Process Engineering, Karlsruhe Institute of Technology, 76344 Eggenstein-Leopoldshafen, Germany; alexander.wunsch@kit.edu (A.W.); paul.kant@kit.edu (P.K.); marijan.mohr@gmx.de (M.M.); katja.haas-santo@kit.edu (K.H.-S.); peter.pfeifer@kit.edu (P.P.)

**Keywords:** membrane reactor, hydrogen, palladium, microstructured, LOHC, suspension plasma spraying

## Abstract

Hydrogen production and storage in small and medium scale, and chemical heat storage from renewable energy, are of great interest nowadays. Micro-membrane reactors for reforming of methane, as well as for the dehydrogenation of liquid organic hydrogen carriers (LOHCs), have been developed. The systems consist of stacked plates with integrated palladium (Pd) membranes. As an alternative to rolled and electroless plated (Pd) membranes, the development of a cost-effective method for the fabrication of Pd membranes by suspension plasma spraying is presented.

## 1. Introduction

Hydrogen is not only a valuable resource for fuel cells and an efficient solution for sustainable mobility. It is used in large quantities for the production of ammonia, alcohols, and fertilizers, and for cracking of heavy crude oil factions and hydrodesulphurization of fuels. Smaller quantities of hydrogen are needed in the electronic and metallurgical industry. Large scale users produce their hydrogen demand on site, mainly via methane steam reforming and purification via pressure swing absorption. Small scale users cannot produce their hydrogen demand economically on site, since the pressure swing absorption does not scale down economically. Therefore, hydrogen is transported to these consumers by truck.

The transportation of hydrogen by truck, usually in compressed or liquefied state, is expensive and energy intensive, due to the low density even of liquefied hydrogen (70 kg/m^3^), and safety aspects on the road. In addition, the transportation, including compression or liquefaction, adds heavily to the carbon dioxide (CO_2_) balance.

A technical innovation must, therefore, be sought in order to supply small consumers of hydrogen—which, after all, require hydrogen consumption of about 5% of the total and, thus, almost 3 million tons per year—through decentralized production. This is not only economically favorable, but also allows the use of bio-gases, instead of natural gas, as a largely CO_2_-neutral hydrogen production which is not burdened by complex transport routes. Compact membrane reformers are an attractive option to produce hydrogen in small to medium size quantities in decentralized locations, on site, for low pressure applications by steam reforming of methane or natural gas. Using microstructured devices, very compact units can be realized. These compact reformers are built of thin metal sheets integrating different functions, such as reforming, separation of hydrogen from the gas by a membrane, and heat integration by internal combustion. The very large ratio of channel surface area to reactor volume leads to good heat and mass transfer properties. The main challenges are the palladium membrane and the integration of the catalyst in the microstructured channels. Advances in the last years in the integration of thinner palladium membranes, to achieve higher efficiency, have been made. Mainly thin supported-metal foils have been employed, also, electroless plating (ELP) is frequently used for laboratory-scale membrane reactors. However, these preparation techniques are expensive, and mostly not suitable for higher quantities, as they are cost-intensive, and manufacturing is time consuming [1].

Besides hydrogen production, the storage of the gas is expensive, due to low volumetric density. In the context of building up the hydrogen infrastructure, a technology is needed that makes storage and distribution lucrative. The use of organic liquids with the ability to bind hydrogen reversibly has great potential in this area. Under certain conditions, and in the presence of a catalyst, the liquid can be hydrogenated and, if required, dehydrogenated again. In loaded or unloaded state, the liquid organic hydrogen carriers (LOHCs) can be transported or stored practically without losses. However, an enormous amount of heat is required for dehydrogenation. In addition, small amounts of unwanted compounds that contaminate the hydrogen are also formed by reaction. Microstructured devices can help out—due to the small dimensions, an almost isothermal reaction management is possible. The use of a Pd-based membrane, enclosed by microstructures, can purify the hydrogen with high efficiency.

In the present article, the concepts for highly integrated compact membrane reformers, the fabrication of thin Pd membranes by suspension plasma spraying, and the application of a membrane reactor concept for the dehydrogenation of liquid organic hydrocarbons, are presented.

## 2. Microstructured Membrane Reactors—µEnhancer 2.0

Several studies and reviews on compact small-scale hydrogen production have been published in the recent years [2,3,4]. Most groups used Pd or Pd alloys as unsupported thin films, or deposited membranes, for in situ hydrogen removal. Rahimpour et al. reviewed applications and preparation methods of palladium membranes. The main preparation methods for Pd-based membranes mentioned by Rahimpour et al. include electroless plating, metal organic chemical vapor deposition (MOCVD), and physical vapor deposition (PVD) plasma sputtering [5], while suspension plasma spraying (SPS), an also promising technique [6], is not mentioned. Fernandez et al. presented a paper on the research at Tecnalia, and TU/e using a fluidized bed membrane reactor concept with methane or biogas as feedstock [7], underlining the feasibility of compact small-scale hydrogen production. In the next chapter, microstructured reactor concepts will be described.

### Designs

All reformer designs developed at the Institute for Micro Process Engineering (IMVT) are modular. Modules with specific functions and production capacity are stacked in a reactor allowing easy adjusting of production capacity.

Based on the first designs of a planar microstructured membrane reactor [8,9], two designs for the second generation of reaction modules were developed at IMVT (µEnhancer 2.0). The first of the two newly developed designs is made up of two modules, one combustion module and one reformer module with integrated hydrogen purification. The second design combines combustion, reforming, and hydrogen purification in one module.

All microstructured modules are built by stacking microstructured stainless steel sheets; the functionalities as gas distribution, reaction channels with catalyst for combustion and reforming, and hydrogen separation via Pd membranes, are realized by the design of the plates and stacks. Laser welding of the stack ensures gas tightness. The hydrogen separation membrane can be incorporated either by laser welding of thin Pd foils, or by coating of porous metallic composite supports with Pd or Pd alloys.

**Figure 1:**
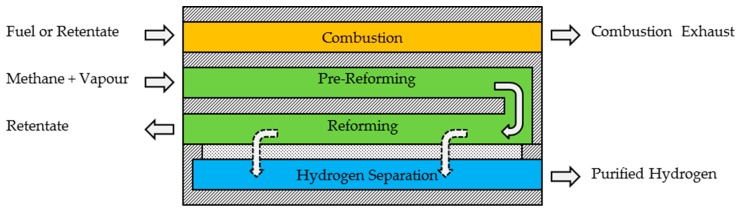
Rough scheme of the combination of the three functionalities (combustion for heat supply, reforming and hydrogen separation) of a fully integrated methane steam reformer in one module.

**Figure 2:**
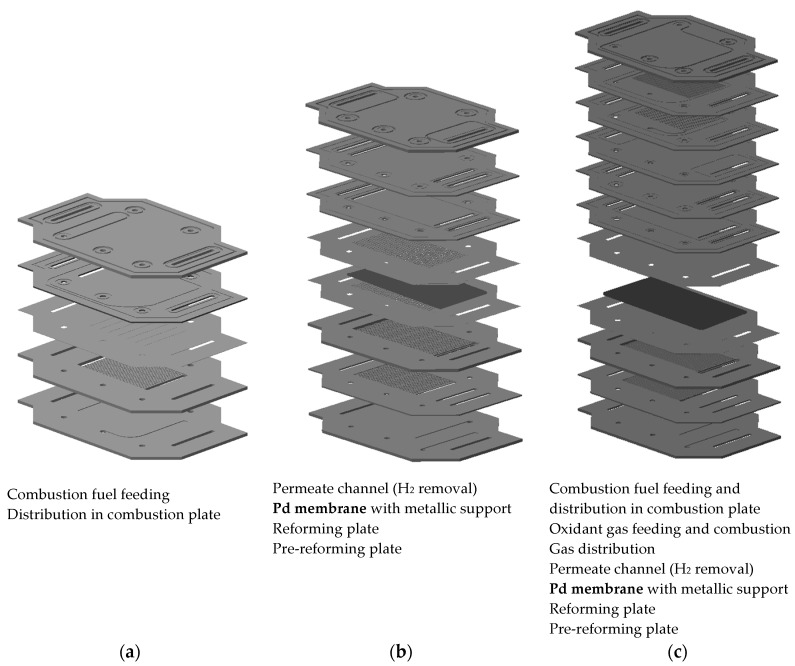
Stack designs for combustion, reformer, and integrated module for the reforming of methane with integrated hydrogen separation: (**a**) combustion; (**b**) reforming; (**c**) integrated.

The main part of the reactors is the reforming of methane. In the µEnhancer 2.0 designs, reforming is carried out in two steps: one pre-reforming step, in which a sufficiently high hydrogen partial pressure for separation is achieved; and one reforming step, in which hydrogen is removed from the reaction channels, simultaneously to the reaction, to push the reaction to higher conversion rates. The heat of reaction for the reforming is supplied via combustion of either additional fuel or the retentate of the reformer layer/module. A comprehensible scheme of the combination of the three functionalities (combustion, reforming, and hydrogen separation) in one module is shown in Figure 1. Detailed schemes of the modules of both newly developed µEnhancer 2.0 designs are shown in Figure 2.

The hydrogen separation part of the stack, shown in Figure 2, is the integral part of both designs. The combustion modules in both designs include a distribution layer for the air needed for combustion, in order to provide uniform heating over the whole area.

In the first design with separated combustion and reforming modules, the process flexibility is quite high, as the temperature can be changed by regulation of the combustion feed gases.

In the second design, a layer for combustion fueled by retentate or new fuel is integrated. This layer is directly adjacent to the reforming, as well as the hydrogen separation by the Pd membrane. This integrated design saves about 15% material and 25% space (height) in comparison to the first design. For comparison of number of plates and stack height, see Table 1.

The challenges in fabrication techniques are high, and the operational flexibility is reduced, and all process conditions for the reactions have to be matched. However, the compactness of this system is very high, and so are the hydrogen yield and productivity for a well-balanced system. An alternative serial arrangement of the functional modules is also possible: first, a microchannel reformer followed by a combined water gas shift membrane reactor, and even these functions can be separated into a microchannel water–gas shift reactor followed by a gas separation unit with a palladium membrane. This modular approach, enabled by micro process engineering, offers important process flexibility, according to the needs present in the distinct setting.

**Figure 3:**
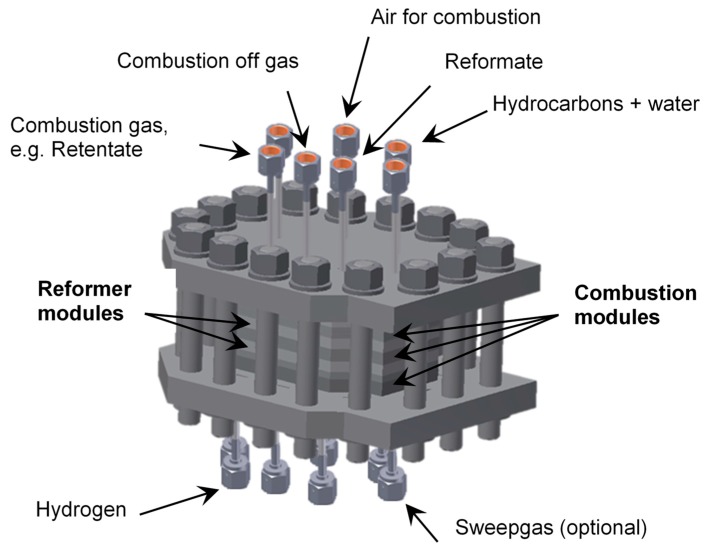
Schematic representation of the modular membrane reactor system with reformer and combustion modules, with inlets and outlets for reaction gases.

The new ultra-compact reactor system for on-site production, in which the described modules are combined/connected to supply tubes, etc., is shown in Figure 3. These reactor systems can be easily integrated in decentralized processes with only small hydrogen demand.

However, for a larger amount of facilities, it is of economic interest to reduce the production costs for the palladium membranes. An approach to substitute the commonly via electroless plating or cold rolling-produced Pd membranes, by ones fabricated by SPS, is presented in the next chapter.

## 3. Palladium Based Composite Membranes via Suspension Plasma Spraying

### 3.1. State of the Art: Membrane Materials

The idea of using palladium as active membrane material for high temperature hydrogen separation goes back more than 100 years. Already in 1916, Snelling patented an “Apparatus for Separating Gases”, which is basically an electroplated palladium membrane on a tubular ceramic support [10]. Meanwhile, a vast number of materials has been proposed as an alternative to the expensive and sensitive palladium, among which are palladium alloys and cermets, group V metals, and alloys containing these metals. Extensive overviews on membrane materials can, for instance, be found in [5,11,12,13]. Palladium is very sensitive towards poisoning, for example, by sulfur species or carbon monoxide, and unstable when submitted to temperature cycles in hydrogen atmospheres crossing the α- to β-phase transition temperature around 293 °C [5,11,12,13,14,15]. However, some palladium-based alloys overcome at least one of these two major drawbacks of pure palladium. Palladium–copper alloys show, for instance, significant tolerance towards sulfur impurities, whereas palladium–silver alloys have lower phase-transition temperatures [11,13,16,17]. In addition, depending on the exact composition, palladium–copper and palladium–silver alloys have slightly higher hydrogen permeabilities than pure palladium, and alloying palladium with cheaper metals makes the membrane material, per mass, less expensive [11,13,14]. Group V metals are also less expensive than palladium, and have impressive hydrogen permeabilities way higher than that of palladium [13,18]. However, they suffer from hydrogen embrittlement and poisoning of active sites for hydrogen dissociation on the surface [12,13,18,19]. Early attempts to overcome surface poisoning of group V metal membranes were made in 1967, when Makrides, Wright, and Jewett patented membranes made from group V metals coated with thin layers of palladium on both sides [20]. Publications from the last years, on group V metal-based hydrogen membranes with palladium-based or transition metal carbide protection layers, show further development but also outline tremendous challenges, among which, attenuation of hydrogen flux over time in consequence of, for instance, interdiffusion of membrane and top-layer materials at elevated temperatures [18,19,21,22]. 

### 3.2. Membrane Design

Beside the choice of the membrane material, the membrane design is crucial when designing a reactor with integrated membrane for hydrogen feed or removal. Free standing membranes like, for instance, fabricated and examined in [18,23], have to be rather thick to be manageable and to withstand pressure differences between the retentate and permeate side. Since membrane materials are expensive, and permeance decreases with increasing thickness, thinner membranes supported on porous substrates are desirable [24,25]. Both ceramic and metallic porous substrates for metallic hydrogen separation membranes are reported in the literature (see e.g., [16,26]). Ceramic substrates have smooth surfaces with small pore sizes and narrow pore size distributions, which facilitate the fabrication of defect-free thin metal layers [24,25,26,27]. Unfortunately, ceramic supports are difficult to integrate into reactors, as joining between metal and ceramic parts is difficult [26]. By contrast, porous sinter metal membrane supports can easily be integrated into reactors, for example, via welding. However, they only show poor surface properties such as wide pore size distributions and large pore sizes [26]. Furthermore, metallic interdiffusion between the metallic support and the active metal membrane layer can deteriorate the membrane performance [24,27].

A promising approach combining both advantages from porous ceramic and metallic supports is, therefore, the fabrication of composite membranes on sinter metal supports with ceramic diffusion barrier layers, like reported, e.g., in [6,28,29,30,31]. Choosing suitable materials for the sinter metal support and the diffusion barrier layer influences the long-term stability of the fabricated composite membrane. Factors such as corrosion resistance at reaction conditions of the porous metal support, and similar thermal expansion coefficients of sinter metal, ceramic, and membrane material, are important. Kot showed that the combination of Crofer-22-APU steel and yttria stabilized zirconia (8 mol % yttria—8YSZ) is, for example, a suitable material combination for the fabrication of substrates for palladium composite membranes [31]. The modules of the µEnhancer 2.0 design are therefore based on Crofer-22-APU as material. The composite support for palladium foils or the sprayed palladium layer is made up of Crofer-22-APU and an 8YSZ diffusion barrier layer.

### 3.3. Standard Membrane Fabrication Methods

There is a variety of techniques reported for the fabrication of metallic hydrogen separation membranes, among which include rolling [18,23,32,33], physical vapor deposition [30], electroplating [28], and electroless plating [28,30,34,35]. Extensive overviews of different fabrication methods are available in the literature (see e.g., [11,36]). Like mentioned above, almost all reported methods are iterative and time-consuming processes, demanding days to weeks to build up sufficiently thin (in the case of rolling) or thick (in the case of physical vapor deposition and electroless plating) metal layers. The most common method to prepare thin (in the range of 10 µm) palladium-based membranes is electroless plating. Using this method, the substrate must be covered with palladium seeds, for instance, via wet chemical methods or physical or chemical vapor deposition. Subsequently, the seeded substrate is immersed in a coating solution containing a stabilized palladium salt, which is then autocatalytically reduced by the palladium seeds under consumption of an added reducing agent. A detailed description of the electroless plating process is, for example, given in [29]. Depending on the properties of the substrate surface, in order to reach a dense defect-free metal layer, the coating procedure must be repeated multiple times [28]. For high surface quality asymmetric ceramic supports, single-step coating procedures for thin membranes are reported, see, for instance, [37]. For the fabrication of palladium alloy membranes, different metals are deposited subsequently, and annealed under elevated temperature (see, e.g., [16,38]). Simultaneous deposition of palladium and silver via electroless plating is also reported, see, for instance, [37,39]. Another drawback of electroless plating, besides the time-consuming iterative layer build up, is the production of metal-loaded wastewater.

A novel and promising technique for the cost-effective fabrication of thin metal-based hydrogen separation composite membranes is under development at IMVT in cooperation with the German Aerospace Centre (DLR) in Stuttgart, and based on industry-established plasma spray techniques. Time requirements to fabricate layers with thicknesses in the 10 µm range lie in the range of minutes. Substrates do not have to be activated like in the case of electroless plating, and no metal-loaded liquid waste is produced [6,30].

### 3.4. Plasma Spraying Techniques for Membrane Preparation

#### 3.4.1. General Process Description

**Figure 4:**
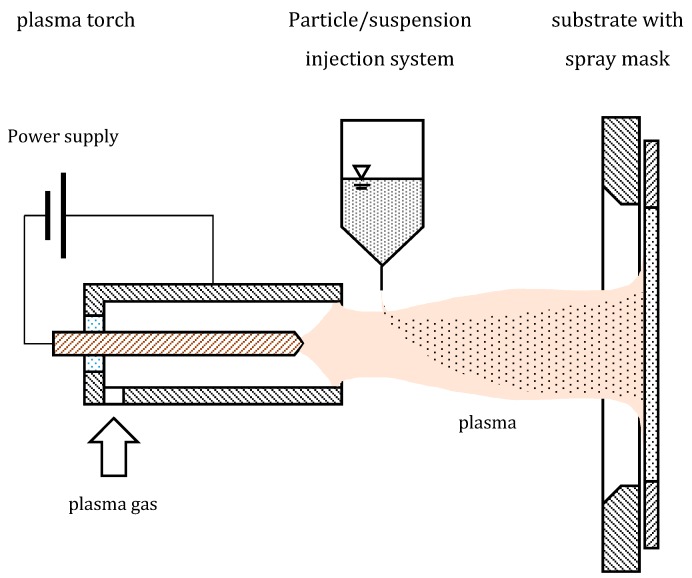
Schematic sketch of a plasma spray facility to produce metallic membrane layers on porous substrates. Plasma is lighted in a plasma torch, and carried out by the plasma carrier gas. Metal particles, or a suspension of metal particles, are injected into the plasma. The molten metal particles hit the substrate surface with high velocity, and form a dense metal layer. Sketch after [6].

Plasma spraying is an industry-established process, which is typically used for the fabrication of thermal barrier layers on gas turbine blades, as well as corrosion and wear protection layers on tube walls and bearings. A schematic sketch of a plasma spray facility is shown in Figure 4. Typically, particles are injected into a plasma of a plasma torch with the help of a carrier gas. The particles injected into the plasma melt and hit a surface, where they form either dense or porous layers, depending on the process parameters. Both ceramic and metal particles can be processed. Large areas can be coated in a short time with impressive deposition rates up to 1 mm/min [40].

If dense layers with thicknesses in the range of only a few microns shall be fabricated, very small particles with mean particle sizes smaller than 1 µm must be injected into the plasma. The injection of such small particles is impossible with a carrier gas, since the particle’s momentum is too small to enable the particles to penetrate the plasma. Instead, suspensions containing the particles are injected into the plasma. The solvent of the injected suspension evaporates when reaching the core of the plasma. The remaining particles melt, and form a layer on the coated surface [41].

Plasma spray processes, and especially the suspension plasma spray process, are very complex, depending on a variety of variables, such as the particle size and agglomeration state of the particles (in the suspension or powder), the solvent used when suspensions are injected, the injection speed and rate, the plasma parameters, such as plasma gas composition and flow rate, plasma current, spraying distance, and spraying atmosphere, and the substrate temperature and surface properties [6,40,41].

#### 3.4.2. Development and State of the Art

Although there are several groups reporting the fabrication of dense membranes via plasma spraying, for example, the fabrication of oxygen ion transport membranes via suspension plasma spraying (see [42]), the authors are only aware of the Dittmeyer group fabricating palladium-based hydrogen separation membranes via plasma spraying. A first attempt was reported 2007, by Dittmeyer and Huang, using atmospheric plasma spraying with a powder feed. They were using a commercial palladium powder with particle size <45 µm and, as substrates, porous stainless steel with plasma-sprayed yttria-stabilized zirconia diffusion barrier layers. The diffusion barrier layers were maintained with their rough surface after spraying. Regarding future prospects, Dittmeyer and Huang argued that a rough surface would improve membrane adhesion. Other groups, using plasma-sprayed ceramic diffusion barrier layers, sand blast the layers to smoothen the surface and facilitate the application of defect-free thin palladium layers (see, e.g., [34]). Despite the thicknesses of 30 µm and 70 µm of the palladium layers deposited by Dittmeyer and Huang, the fabricated composite membranes had no satisfactory permselectivity, due to remaining open porosity. Dittmeyer and Huang wrote that smaller particles injected into the plasma could result in thinner and denser metal layers. [30]

A second attempt at fabricating palladium composite membranes via plasma spraying techniques was reported 2014 by Boeltken et al. Like Dittmeyer and Huang, Boeltken et al. were using porous stainless steel substrates with sprayed yttria-stabilized zirconia diffusion barrier layers. The surface of the diffusion barrier layers in the experiments of Boeltken et al. also remained in its rough initial state. Palladium particles, with a size between 250 nm and 550 nm, were injected into the plasma dispersed in dethylene glyocol monobutyl ether stabilized with ethyl cellulose. Boeltken et al. fabricated much thinner layers of palladium than Dittmeyer and Huang (9.5 µm instead of 30 µm and 70 µm, respectively) but the permselectivity of the fabricated composite membranes was still not satisfactory; it only reached a value of 60. For the future, Boeltken et al. proposed to use substrates with smoother surfaces and smaller pore sizes to reach higher permselectivities at smaller palladium layer thicknesses [6].

**Figure 5:**
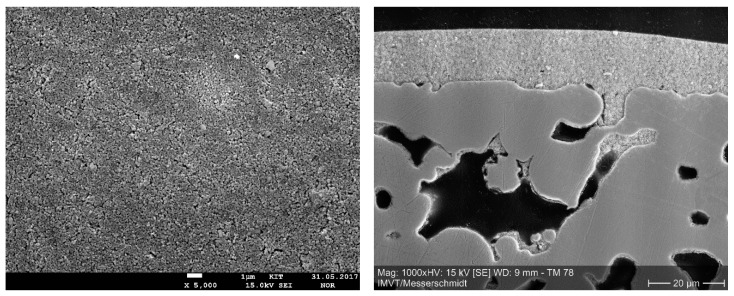
Surface (**left**) and cross-sectional view (**right**) of substrates currently fabricated at IEK-1 in Jülich and Institute for Micro Process Engineering (IMVT) in Karlsruhe.

In 2017, a third attempt to fabricating palladium-based composite membranes for hydrogen separation started in the cooperation network of the Institute for Energy and Climate Research (IEK-1) at Research Centre Jülich (FZJ), DLR, in Stuttgart, and IMVT in Karlsruhe. Since the substrate surface quality was of great importance in the experiments of Boeltken et al., the project focused, at first, on the fabrication of high-quality composite membrane substrates. Detailed results of the substrate fabrication and characterization, conducted in 2017, are reported in [43]. Crofer-22-APU sinter metal plates, provided by IEK-1, were welded into dense metal frames for good integrability into test reactors, and coated via dip-coating with an yttria-stabilized zirconia layer. The resulting membrane substrates had a smooth defect-free surface. Figure 5 shows scanning electron microscope (SEM) images of the surface, and a cross-section of the fabricated substrates. The surface-weighted pore size distribution was determined from SEM images, and followed an approximately log–normal distribution, with a geometric mean pore size of 98 nm, and a geometric standard deviation of 0.617. The nitrogen permeance at room temperature of the substrates was determined to be 7.9 ± 1.2 µmol/m^2^/s/Pa. Hydrogen permeance at room temperature was 22 ± 3 µmol/m^2^/s/Pa. The literature reports substrates with room temperature nitrogen permeances more than twice as high as the value determined in the current work at IMVT (see e.g., [28]). Further investigations showed that the main transport resistance in the fabricated substrates did not lie in the ceramic diffusion barrier layer, but in pores of the sinter metal substrates blocked with coating suspension formed during dip-coating. This shows a potential for improvement of the permeance of the substrates. The palladium layer was deposited at DLR in Stuttgart via suspension plasma spraying. One focus of the coating process development was the fabrication of stable palladium suspensions departing from commercially available palladium powder with a mean particle size of ca. 100 nm. First, results were not yet satisfactory, since the suspension stability and deagglomeration state of palladium particles in suspension were not optimal, and the deposited layers were very thin (1–4 µm) and with a remaining open porosity, but work is ongoing.

## 4. Process Intensification in LOHC Dehydrogenation Using Pd-Based Membranes

Liquid organic hydrogen carriers are recently discussed substances that could give an answer to the question of safe and handy storage and distribution of hydrogen [44,45]. Especially with regard to future energy systems fed from renewable sources, the suitability of this technology will be investigated [46,47]. The hydrogen is produced via electrolysis supplied by regenerative electricity to provide further applications independent of location and time. The extraction of hydrogen in locations, where a lot more hydrogen is available than needed, makes this technology feasible. These locations are mostly sunny and windy regions or places where no customers can be found. Conventional solutions store hydrogen physically, by compression or liquefaction, to increase the low volumetric energy density compared to atmospheric conditions. LOHC technology, on the other hand, propagates the reversible chemical binding of hydrogen to an organic liquid. Due to a high cycle stability, the use of the carrier substance should resemble a “H_2_ deposit bottle”. A suitable LOHC is also characterized by high temperature stability, low toxicity, high storage density, good availability and, ultimately, thermodynamic conditions that enable profitable technical implementation. The decisive advantage of this technology lies in the secure and long-term stable storage, as well as the use of existing infrastructure for the distribution of the liquid. A comparison of the mentioned storage technologies and the potential LOHC material systems is discussed, in detail, by Preuster et al. [48].

Brückner et. al [49] have shown that the isomer mixture of dibenzyltoluene (0H-DBT, Marlotherm SH^®^ from Sasol), which is commercially available as a heat transfer oil, partially or completely fulfils all these criteria and is, therefore, suitable as LOHC. The LOHC can store 6.2 wt % hydrogen when fully loaded, which corresponds to a storage density of 17.5 L_LOHC_/kg_H2_. A study on environmental and health impact attributes a high potential of acceptance in the population by the proposed material, since the handling of organic liquids, such as diesel, is already known [50]. Furthermore, 0H-DBT and the hydrogenated form, perhydro-dibenzyltoluene (18H-DBT), have a considerably lower risk potential than fuels used today—they are neither flammable nor volatile. In addition, no carcinogenic effects have been proven [51,52]. The system 18H-DBT/0H-DBT is, therefore, currently the state of the art in LOHC research. Various applications, considering 18H-DBT/0H-DBT as carrier system, were evaluated on a thermodynamic and techno-economic basis [53,54,55]. Rüde et al. predict high reliability and robustness to the LOHC unit with upstream electrolysis and a downstream fuel cell [5,56]. Dynamic operation of a dehydrogenation unit has already been demonstrated [57].

In order to be able to describe the substance system, which has not yet been sufficiently researched for this purpose, firstly, material properties had to be determined [58,59,60,61,62]. Secondly, suitable analytics had to be found to determine the degree of (de)hydrogenation. A comparison of different methods has shown that refractometry is the simplest, and nuclear magnetic resonance (NMR) spectroscopy the most accurate method to determine the hydrogen load of the carrier [63,64]. Recent developments show that, despite high boiling points (0H-DBT ≈ 390 °C) of the substances, gas chromatography can also be used [65].

Considering thermodynamics, favorable conditions result for hydrogenation at high pressures (15–30 bar) and low temperatures (150–250 °C), and for dehydrogenation at low pressures (1–5 bar) and high temperatures (280–330 °C). Consequently, the superimposed phase and reaction equilibrium can only be determined with great inaccuracies in these multiphase reactions [66]. Do et al. investigated the reaction path, and found that the reaction takes place via two stable intermediates (12H-DBT, 6H-DBT) resulting from consecutive C6-cycle dehydrogenation [67]. In total, the 0H-DBT/18H-DBT can absorb/release nine H_2_ molecules, whereby 71 kJ/mol_H2_ heat is released/consumed per molecule [49]. The strong heat tone means that, in case of decentralized applications, a lot of waste heat is needed. Therefore, Preuster et al. investigated the power generation by linking the dehydrogenation unit with a solid oxide fuel cell [68].

In general, dehydrogenation is technically more difficult to implement than hydrogenation. In addition to the thermodynamically unfavorable conditions, the enormous gas production makes the reaction control more difficult. Assuming that the residence time of the gas and liquid phase is equal, the reaction chamber is almost exclusively filled by gas. Depending on the reactor concept, the liquid can be “pressed” through the apparatus by gas bubbles with short contact times at the catalyst. Including wetting properties between catalyst and LOHC, this flow guidance can lead to enormous losses in catalyst-related productivity. The high gas content additionally causes LOHC to evaporate due to the vapor–liquid equilibrium (VLE), which is detectable despite subsequent condensation of the product gas. In addition to traces of LOHC, other low-boiling components could also be determined, which result from side reactions, e.g., from decomposition or transfer hydrogenation [69]. In order to provide hydrogen in sufficient quality for other applications, such as fuel cells, purification of the product gas is, therefore, still indispensable.

### 4.1. State of the Art: LOHC Dehydrogenation

Hydrogenious Technologies GmbH, a young company from Erlangen, Germany, offers commercial container-based solutions for loading/unloading liquid hydrogen carriers in different performance classes [52]. As reactor geometry for dehydrogenation, a horizontal pipe, half-filled with catalyst particles, is used. The liquid flows in from below and covers the catalyst. This allows the resulting hydrogen to be removed from the top [70]. This approach prevents a multiphase flow, and allows the liquid to almost follow the hydrodynamic law.

Another concept is the so-called “one reactor”, which makes it possible to switch between hydrogenation and dehydrogenation reactions in the same container with the same catalyst and similar temperature level, purely by changing the reaction pressure. A benefit, especially for stationary applications, is the possibility to recuperate the heat obtained from the hydrogenation reaction. In addition, investment costs are significantly reduced. The reactor consists of a vessel with vertically arranged tubes filled with catalyst, which are tempered from the outside by a thermostat. In the dehydrogenation configuration, the loaded LOHC flows from bottom to top, so that the resulting gas bubbles leave the pipes at the top outlet [71].

Both concepts presented, however, cannot deliver high-purity hydrogen, due to low-boiling by-products and traces of evaporated LOHC. A purification can be managed, e.g., by a pressure swing adsorption plant (PSA) which, however, becomes unprofitable with low hydrogen capacities. One solution to this problem could be the integration of Pd-based membranes as a purification step.

### 4.2. Multi-Staged Approach Using Pd Membranes

**Figure 6:**
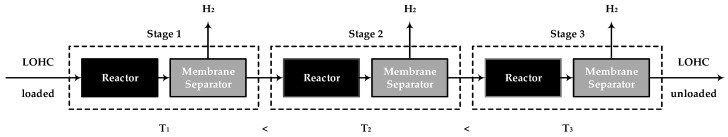
Schematic of the multi-staged reactor concept with intermediate hydrogen separation.

Pd-based membrane reactors have already been developed for the dehydrogenation of LOHC methylcyclohexane, and the steam reforming of methane, where promising results could be achieved [3,8,9,72,73,74,75]. For adaptation to the multiphase system 18H-DBT/0H-DBT/H_2_, a multi-stage reactor concept with intermediate separation of hydrogen was developed. The use of consecutive reactors and membrane separators is not new, however, the concept has so far only been applied for reactions in the gas phase. Due to the unknown interaction between LOHC and the membrane surface, the integrated form, the membrane reactor, was avoided in this work. In order to shed light on the influence of the intermediate separation, a reactor and a membrane separator were modeled and sequentially arranged (see Figure 6). The simulations were carried out under strong simplified conditions, in order to show the positive influence of the gas separation via Pd-based membranes from the system. For the reactor, a radial flow reactor was assumed, which behaves like a plug flow reactor (PFR). This is a 1-dimensional problem which was solved integrally by the CSTR (continuous stirred tank reactor) cascade using MATLAB software. The resulting product stream from the reactor stage is fed to the membrane separator, where the hydrogen is completely separated. This also means that the potential wetting of the membrane surface by LOHC, which leads to a reduction of the effective membrane area, has not been taken into account. For the calculations, no experimentally determined kinetics were used for the reaction—rather, the kinetics were adjusted such that a relevant degree of dehydrogenation could be achieved. A detailed description of the modeling, the reactors used, and the experimental determined kinetics of the system, can be found in [Wunsch 2018, manuscript will be submitted for the special issue].

To characterize the reactor, the ratio of the residence time between the gas phase and the liquid phase must first be defined. The least efficient case was assumed, which means that the arising gas phase stays the same time as the liquid phase:

(1) $$\;\tau_{liq}=\tau_{gas}.\;$$

In order to describe the influence of the high gas production on the effectively used catalyst mass, a correction factor, α, will be introduced:

(2) $$\;m_{Cat}^{eff}=\alpha \cdot m_{Cat}.\;$$

The correction factor primarily describes the poorer wetting of the catalyst with LOHC under the condition that the gas and liquid phases have the same residence time. For a simplified description of this property, a relationship between correction factor α and liquid phase fraction $\varepsilon_{L}$ was constructed:

(3) $$\;\alpha =\ln\left(1+\varepsilon_{L}^{b}\cdot \left(e-1\right)\right).\;$$

**Figure 7:**
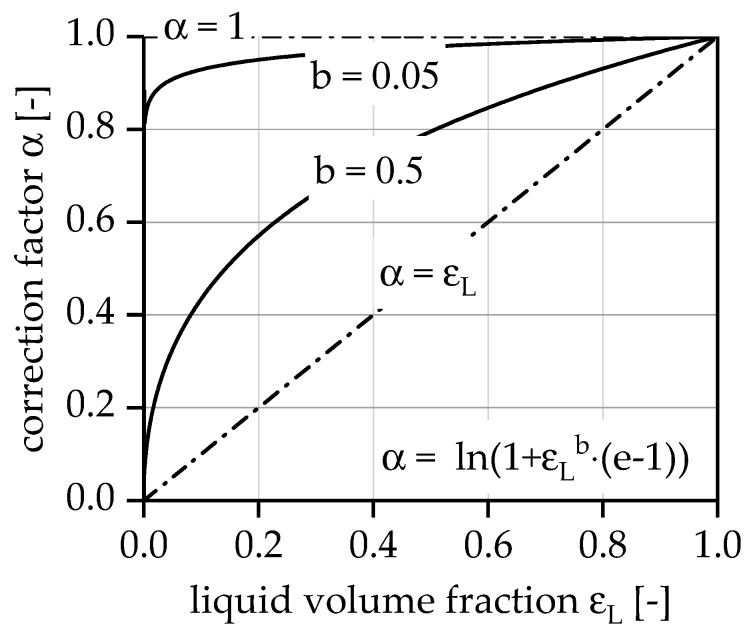
Constructed relationship between correction factor α and liquid volume factor$\;\varepsilon_{L}$.

This relationship is not determined experimentally; it is simply a construction that could describe the unknown efficiency loss by reduced wetting of the catalyst surface area with decreasing liquid phase fraction in the reactor. As the conversion increases, the liquid phase fraction decreases and, finally, the effectively used catalyst mass decreases (see Figure 7). This results in the limiting case α=1, when the evolved gas has no impact on the efficiency, and the limiting case $\alpha \approx \varepsilon_{L}$, when the efficiency decreases nearly linear with gas production. This relationship can be controlled using parameter b.

As mentioned, we assumed that an increased degree of dehydrogenation is present in each reactor stage, and that the membrane completely separates the hydrogen from the product gas:

(4) $${\dot{V}}_{H2,\;Retentate}=0.$$

**Figure 8:**
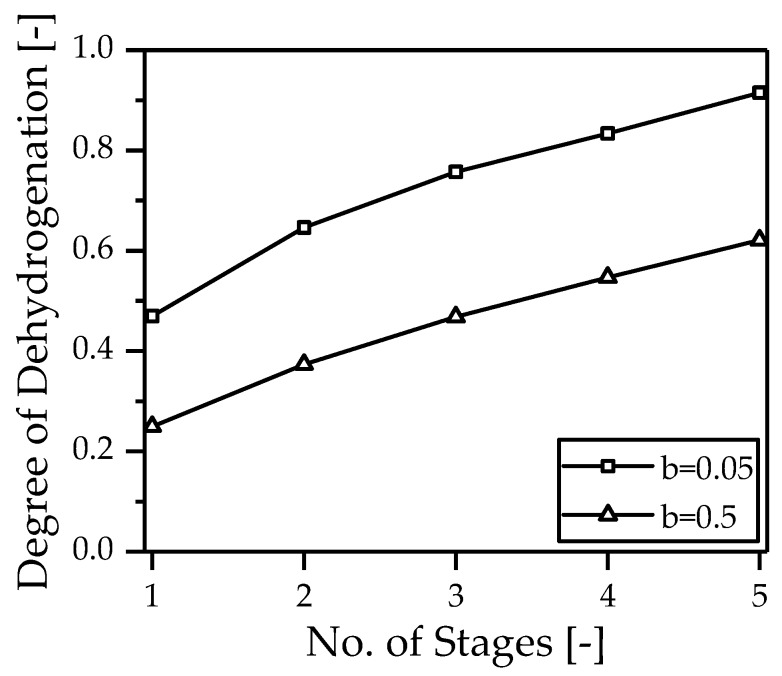
Degree of dehydrogenation as a function of the number of reactors used with intermediate separation at constant catalyst mass and varying influence of the gas phase on the effectively used catalyst mass.

Figure 8 shows the degree of dehydrogenation depending on the number of reactors used. A constant catalyst mass is distributed over a different number of reactors (1–5). A reactor is divided into N equal pieces, and the hydrogen is removed at these points via a Pd-based membrane. It is immediately apparent that the degree of dehydrogenation can be almost doubled by the more frequent intermediate separation alone, irrespective of the residence time distribution between gas and liquid phase. As expected, the total degree of dehydrogenation increases with decreasing impact of the gas phase on the effectively used catalyst mass (b=0.05).

**Figure 9:**
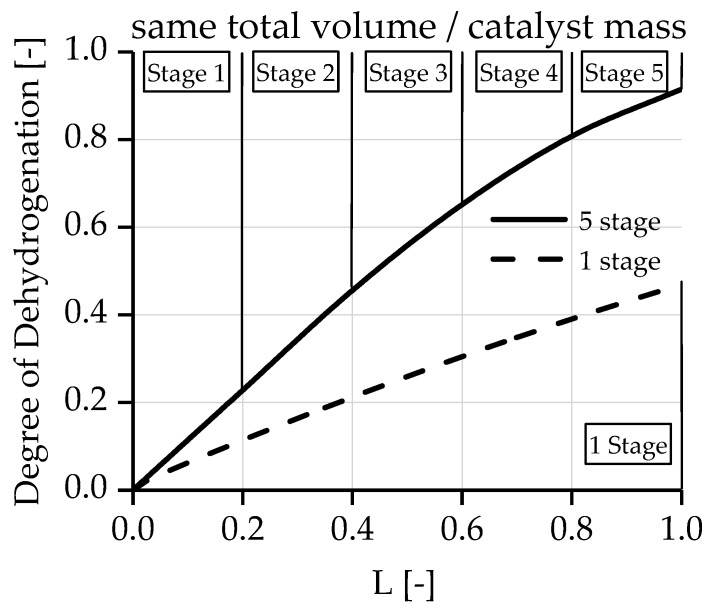
Dehydration degree over the reactor length with different number of separation stages.

A comparison of the degree of dehydrogenation between five reactor stages, with intermediate separation and one reactor stage, is shown in Figure 9. The reactor volume/catalyst mass was also kept constant. It can be seen that already after 40% of the reactor length, and one intermediate separation stage, the same degree of dehydrogenation is achieved as after the entire reactor length without separation.

It can be summarized that the multi-staged reactor concept with intermediate hydrogen separation can be used to intensify the process of LOHC dehydrogenation of 18H-DBT. In addition to the actual goal, namely, hydrogen purification, the intermediate separation increases the efficiency of the process by reducing the residence time of the liquid.

## 5. Conclusions

In this article, we described the general concept for ultra-compact microstructured membrane reactors for hydrogen production. The benefits of the reactor concept are the very large membrane surface area per catalyst volume (ca. 103–106 m^−1^) and negligible mass transport resistance towards membrane, even for high-flux membranes. By the integration of the heating module in the compact device, efficient heating by hot gas or catalytic combustion of retentate with air is ensured. Altogether, the design convinces by its high compactness, low weight, and modular plant design.

Furthermore, an alternative method, suspension plasma spraying, for the fabrication of Pd membranes in relation to the commonly used membranes, either thin metal foils or layers fabricated by electroless plating, has been shown. As supports, porous sinter metallic supports made of Crofer-22 APU with a YSZ diffusion barrier layer have been used. An important advantage of the Crofer-22 APU supports is the possibility for gas-tight integration into the compact module by laser welding.

As an example for the application of these compact modules, besides on-site methane reforming (see [8]), the dehydrogenation of liquid organic hydrogen carriers is described. Here, the advantages, like the equilibrium shift of the reaction and the integrated purification of the released hydrogen, are presented in a multi-staged reactor concept. This concept can be realized in a single compact device by stacking of multiple combined reaction and separation modules.

One important aspect of the application of compact membrane reactors for the catalytic reaction are suitable preparation methods for the catalyst in the microstructured reactors. These developments and results of the preparation of catalyst layers by inkjet printing in the thickness of 10–15 µm are not in the focus of this article, being reported elsewhere [8,76]

Looking ahead, the first commercial applications of membrane reactors may appear in small-capacity hydrogen production for industrial uses via on-site reforming, where hydrogen is at a relatively low pressure (<3 bar) and moderate purity (max 99.5%), and for hydrogen generation from LOHC in the context of hydrogen logistics, rather than in large-scale reforming or water gas shift (WGS). This modular concept is a flexible concept for a variety of applications producing hydrogen, like in reforming reactions and LOHC dehydrogenation. Small-capacity hydrogen supply is an excellent opportunity for the extension of new technologies, such as compact membrane reactors, as they are more energy-efficient compared to large-scale processes, and require a lower OPEX (operating expenditure), and by the simplification of the process scheme, a reduced CAPEX (capital expenditure) is needed. However, for the commercialization of this technology, there is some indispensable research work that is still ongoing at our institute.

## Figures and Tables

**Table 1:** Height and number of plates of single and combined modules.

	Combustion	Reforming	Stacked (Comb. + Ref.)	Integrated
Plates	5	8	13	11
Height	7.2 mm	9.4 mm	16.6 mm	12.4 mm
